# Multifocal musculoskeletal tuberculosis mimicking multiple bone metastases: a case report

**DOI:** 10.1186/s12879-016-1376-7

**Published:** 2016-01-29

**Authors:** Meiping Ye, Jinwei Huang, Jie Wang, Jianmin Ren, Jianfei Tu, Weibo You, Taohui Zhu

**Affiliations:** 1 Fifth Affiliated Hospital of Wenzhou Medical University, Lishui, China; 2 Institute of Antibiotics, Huashan Hospital, Fudan University, Shanghai, China

**Keywords:** Multifocal musculoskeletal tuberculosis, Bone metastases, Atypical presentation

## Abstract

**Background:**

The occurrence of non-contiguous, multiple, and remote involvement tuberculous spondylitis is rare. The clinical presentation in patients with multifocal musculoskeletal tuberculosis may closely mimic that in patients with multiple bone metastases, which makes the accurate clinical diagnosis challenging. Herein, we report a multifocal musculoskeletal tuberculosis case that was misdiagnosed for 8 months as multiple bone metastases.

**Case presentation:**

A 63-year-old male farmer of Chinese Han ethnicity presented to us with pain in left side of the neck, right side of the chest and the back for 10 months without typical tuberculosis symptoms. His past medical history, the CT and fluoroscopy-guided biopsy were negative for tuberculosis. Interferon gamma by T-SPOT was also negative. Radiological findings including CT, MRI and PET-CT suggested that the patient had multiple metastases. Accordingly, the patient was misdiagnosed as having musculoskeletal tumors until a swelling under the right nipple ulcerated. The smear test for acid-fast bacilli and the PCR test for TB-DNA of the pus from the swollen area were both positive, leading to the final correct diagnosis of musculoskeletal tuberculosis.

**Conclusion:**

The proper diagnosis of musculoskeletal tuberculosis is clinically challenging due to *Mycobacterium tuberculosis* variants involved and atypical presentations, especially when the lesions are multiple. Our findings indicate that multiple tuberculous spondylitis must be considered in the differential diagnosis of multiple musculoskeletal lesions.

## Background

*Mycobacterium tuberculosis*, the causative agent of tuberculosis (TB), is a serious pathogen in many parts of the world. According to the World Health Organization, one-third of the world’s population, almost 2 billion people, is infected with *M. tuberculosis* [1, 2]. TB is a concern even in the developed countries; there were 65 TB outbreaks in the United States between 2002 and 2011 that were investigated by the CDC [3]. *M. tuberculosis* usually affects the lungs [4]. In addition, bones and joints can also be affected (skeletal TB, which accounts for 5 % of all cases). Among these cases, spines are most frequently affected and account for almost 50 % of diagnosed skeletal TB [5].

In the case of spinal tuberculosis, two or more contiguous vertebrae are usually involved due to hematogenous spread of bacteria since one vertebral artery feeds two adjacent vertebrae. As a result, most of the reported cases of spinal tuberculosis have lesions that only involve two adjacent levels [6]. According to reported data, the incidence of multiple-level non-contiguous vertebral tuberculosis is 1.1 % to 16 % of all skeletal TB cases [7]. Here, we present a case of multifocal musculoskeletal tuberculosis involving multiple levels of the spine that was misdiagnosed as musculoskeletal tumors for 8 months.

## Case presentation

A 63-year-old male farmer of Chinese Han ethnicity presented pain in his left neck, right chest wall and back for 10 months, along with swelling under the right nipple for 1 month. He was admitted to the hospital on November 26, 2014. The left neck pain with no particular predisposing factors was dull and intermittent in the beginning. It gradually progressed and extended to the left upper extremity, where the pain was more serious and accompanied by numbness at night. The pain eventually spread to the chest and back. The patient has no history of exposure to TB or any recent weight loss, cough, low-grade fever, decreased appetite or night sweats.

**Fig. 1:**
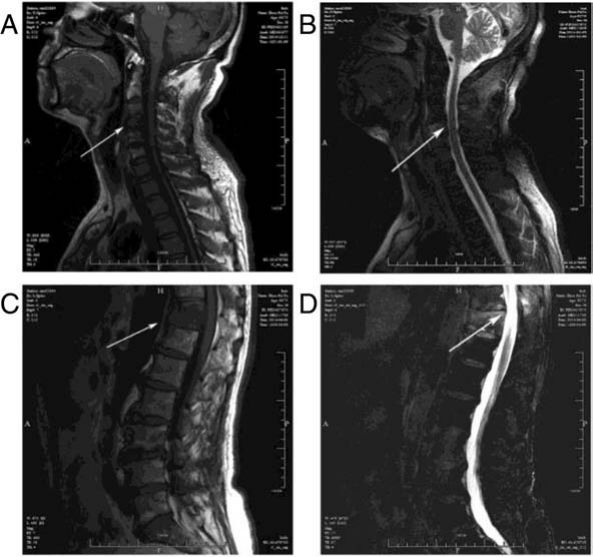
MRI image of the patient with multifocal musculoskeletal tuberculosis. **a** MRI showed degeneration in cervical vertebrae. T1-weighted sagittal image showed heterogeneous, mostly hypointense, lesions involving the C4 and C5 vertebrae (*arrowhead*). **b** T2-weighted sagittal image showed posterior protrusion of C3-4, C4-5, C5-6 and C6-7 and light compression of the corresponding meninges capsule (*arrowhead*). **c** MRI showed degeneration of thoracic vertebra. T1-weighted sagittal image showed heterogeneous, mostly hypointense, lesions involving T11 (*arrowhead*). **d** T2-weighted sagittal image showed posterior protrusion of T11 and light compression of the corresponding meninges (*arrowhead*)

During the previous 10 months, the patient had been hospitalized first in the department of orthopedics and then in the department of interventional radiology. Enhanced chest computed tomography (CT) studies did not show any parenchymal lung lesions or lympho-adenopathy. Then, CT and magnetic resonance imaging (MRI) both showed multiple osteolytic bone lesions at C4, C5, C6, T10 and T11 vertebrae, which were noted along with similar lesions on the ribs of these vertebrae. The dural sac was pressed at the corresponding surface. A bone marrow biopsy revealed granulocytosis in the bone marrow. A fine needle aspiration biopsy of the 9th rib lesion showed that both bones and cartilages had a small amount of fibrous tissue attached to them. Accordingly, multiple bone metastases were suspected (Fig. 1). Final confirmation of the diagnosis was not performed, and the patient was discharged from the hospital with a prescription of morphine sustained-release tablets to relieve the pain. The diagnosis of the patient could not be confirmed by several hospitals for the following 8 months and no additional biopsies were performed.

The patient was re-admitted on November 26, 2014 as the conditions worsened with the development of numbness in both legs. A red swollen area appeared on the right side of the chest without obvious predisposing factors 1 month before re-admission. The swelling with obscure boundary and poor mobility grew gradually from the initial size to about 1 cm × 1 cm at the time of re-admission.

The vital signs of the patient were stable at the time of re-admission. No obvious abnormal signs were observed in neurologic examination. Laboratory results were as follows: Routine blood test: WBC 11.7 × 10^9^/L, N 75.8 %, RBC 3.06 × 10^12^/L, HGB 89 g/L, PLT 232 × 10^9^/L, and serum albumin 38 g/L. Serum protein electrophoresis and immune fixation electrophoresis were normal. The erythrocyte sedimentation rate (ESR) and C-reactive protein (CRP) level were 60.08 mm/hr (normal value 0–15 mm/hr) and 37.55 mg/L (normal value 0–8 mg/L), respectively. Serum tumor markers including AFP, CEA, CA199, CA724 and PSA, were all within normal ranges. Interferon gamma by T-SPOT was negative. Hepatitis B surface antigen (HBsAg) and HIV were negative.

**Fig. 2:**
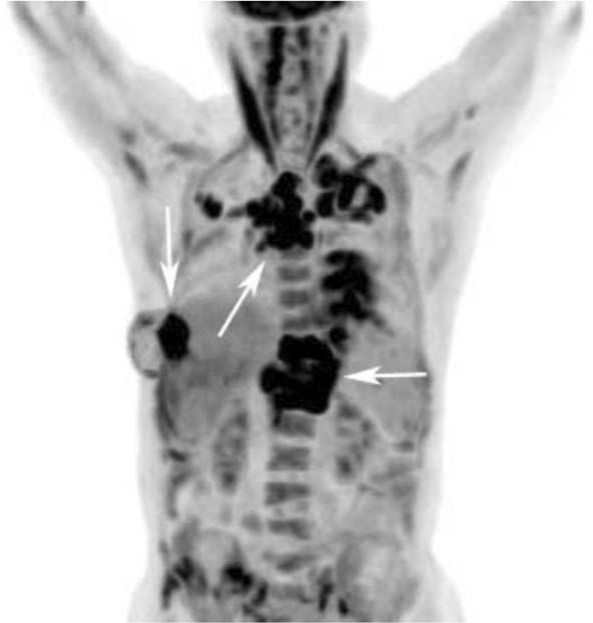
CT image of the patient with multifocal musculoskeletal tuberculosis. F-18 FDG-PET/CT images showed irregular increased FDG uptake (*arrowheads*) with maximum SUVs ranging from 10.2 to 18.5 and involving bilateral collarbones, ribs, thoracolumbar vertebrae, ilium, sacrum, pubis, hip and the left side of the femoral neck

A smear test for acid-fast bacilli of pleural effusion was negative on November 27, 2014 (CT didn’t show pleural effusion in April, 2014). A routine pleural effusion test showed: yellow, turbid, WBC: 1500/μl (neutrophils 55 %, lymphocytes 45 %), and RBC: 1550/μl. Rivalta’s test was positive. Pleural effusion biochemical examination showed: total protein 48.3 g/L, lactate dehydrogenase 188 U/L, adenosine deaminase 12.7 U/L, and glucose 7.08 mmol/L. Malignant cells were not detected in the pleural effusion. 18 F-fluoro-2-deoxy-D-glucose positron emission tomography (FDG-PET)/computed tomography (CT) revealed that there were multiple metastases in the bilateral pleural effusion, whole bone, bilateral hilar lymph nodes, mediastinal lymph node and chest wall (Fig. 2). But a primary tumor location was not detected.

On December 2, 2014, the swelling soft tissue under the right nipple ulcerated. Both a smear test of acid-fast bacilli and a polymerase chain reaction (PCR) of TB-DNA of the pus were positive. As a result, the diagnosis was corrected to multiple musculoskeletal tuberculosis. After 1 month of anti-tuberculosis combination therapy (rifampicin, isoniazid, pyrazinamide and ethambutol), the swelling significantly decreased and the back pain was reduced. After 3 months of treatment, the numbness in the arms resolved.

## Discussion

Although tuberculosis has drawn great attention, it is still a major worldwide health burden. The incidence of musculoskeletal TB appears to be increasing [8, 9]. In spite of the availability of diagnostic modalities, surgical techniques and effective anti-tuberculosis regimens, musculoskeletal tuberculosis (especially spinal tuberculosis) remains a life-threatening disease that can cause bone destruction, deformity and neurological defects [10]. The case presented here was misdiagnosed as a musculoskeletal tumor due to the negative results of the T-SPOT test and the pathological reports, as well as the abnormal displays in the MRI and PET-CT imaging. Looking back on this case, we found that several points should have been considered by the clinicians to facilitate the correct diagnosis.

First, pain is the main symptom in bone tuberculosis [10]. Fever and systemic symptoms may not present until the late stages of musculoskeletal tuberculosis. Thus, pain may persist long before a definite diagnosis [11]. So, clinicians should keep in mind the possibility of musculoskeletal tuberculosis when patients present long-term musculoskeletal pain even without fever or any other systemic symptoms.

Second, bone or soft tissue biopsies may be very important to make a definite diagnosis. However, we should bear in mind that because of the low bacterial load in musculoskeletal tuberculosis, the possibility of detecting *Mycobacterium* may be less than 50 % [12]. Multiple biopsies should be performed and more time and attention should be given to microscopy to improve the rate of correct diagnosis of *M. tuberculosis*.

Third, a negative T-SPOT result was also a contributing factor to the delayed diagnosis. Individuals suspected of having tuberculosis showed a sensitivity of 69–83 % and specificity of 52–61 % for T-SPOT [13]. T-SPOT cannot distinguish latent tuberculosis from active tuberculosis, and it is not overly specific for active tuberculosis [14, 15]. PCR has been recently suggested as an accurate diagnostic tool since both its sensitivity and specificity are high, especially for specimens from a sterile area [16].

Forth, as shown in this case, the slightly increased leukocyte count has a limited value in diagnosis [10]. Increasing CRP and ESR serum levels were reported as potential tuberculosis markers in patients with (−) sputum AFB [17, 18]. CRP was especially emphasized to be a good marker for indicating a response to the anti-tuberculosis treatment. Although the specificity of CRP and ESR are low to diagnose tuberculosis, we should emphasize their diagnostic value. [17].

Finally, imaging examination is not sensitive for differentiating multiple bone metastases from multiple musculoskeletal tuberculosis. MRI is more sensitive for the diagnosis of musculoskeletal tuberculosis. However, when the tuberculosis involves multiple, non-contiguous vertebrae, the imaging appearance can be easily misjudged as metastatic malignancy [19]. FDG-PET plays an important role in differentiating benign from malignant tumors, and in the staging and follow-up based on the intensity of FDG uptake [16]. In addition to tumor tissues, the FDG tracer also accumulates in sites of infection and inflammation. The active tuberculous lesion sometimes consists of epithelioid cells, langhans giant cells and lymphocytes. As such, cells in the active tuberculous lesion exhibit increased glucose metabolism and intense FDG uptake, which can cause misdiagnosis when differentiating tuberculosis from malignancy [20]. Since FDG-PET is not tumor-specific, microbiological and histopathological examinations may be necessary to reduce the possibility of image misdiagnosis.

## Conclusions

Atypical presentations of multiple musculoskeletal tuberculosis pose diagnostic challenges. Mutiple involvement of tuberculous spondylitis must be considered in the differential diagnosis of multiple musculoskeletal lesions. Microbiological and histopathological examination may be necessary to reduce the possibility of image misdiagnosis.

## Consent

Written informed consent was obtained from the patient for publication of this case report. A copy of the written consent is available for review by the Editor of this journal.
